# A Homozygous* CASQ2* Mutation in a Japanese Patient with Catecholaminergic Polymorphic Ventricular Tachycardia

**DOI:** 10.1155/2019/9056596

**Published:** 2019-01-08

**Authors:** Taishi Fujisawa, Yoshiyasu Aizawa, Yoshinori Katsumata, Akihiro Udo, Shogo Ito, Kazumasa Hatakeyama, Makoto Hirose, Hiroshi Miyama, Kazuaki Nakajima, Takahiko Nishiyama, Takehiro Kimura, Masamitsu Nitta, Kazuo Misumi, Seiji Takatsuki, Kenjiro Kosaki, Keiichi Fukuda

**Affiliations:** ^1^Department of Cardiology, Keio University School of Medicine, Tokyo, Japan; ^2^Department of Cardiology, Chiba Nishi General Hospital, Chiba, Japan; ^3^Center for Medical Genetics, Keio University School of Medicine, Tokyo, Japan

## Abstract

A 62-year-old female had suffered from recurrent syncopal episodes triggered by physical and emotional stress since childhood. She had no family history of sudden death. An intensive examination could not detect any structural disease, and exercise stress testing provoked polymorphic ventricular ectopy followed by polymorphic ventricular tachycardia accompanied with syncope leading to a diagnosis of catecholaminergic polymorphic ventricular tachycardia (CPVT). A genetic analysis with a next generation sequencer identified a homozygous W361X mutation in the* CASQ2* gene. Careful history taking disclosed that her parents had a consanguineous marriage. Here we present a Japanese patient with a recessive form of CPVT.

## 1. Introduction

Catecholaminergic polymorphic ventricular tachycardia (CPVT) is an inherited primary arrhythmia syndrome, which is characterized by adrenergic induced bidirectional or polymorphic ventricular tachycardia [1]. A recessive form of CPVT is caused by homozygous or compound heterozygous mutations in the calsequestrin 2 gene (*CASQ2*) [2]. The incidence of* RyR2* mutations in CPVT is about 65%, whereas mutations in the* CASQ2* gene account for only 1% [3]. An autosomal dominant form of CPVT is caused by mutations in the cardiac ryanodine receptor gene (*RyR2*). Although several compound heterozygous* CASQ2* mutations have been reported, homozygous* CASQ2* mutations are scarcely identified in CPVT patients.

## 2. Case Report

**Figure 1:**
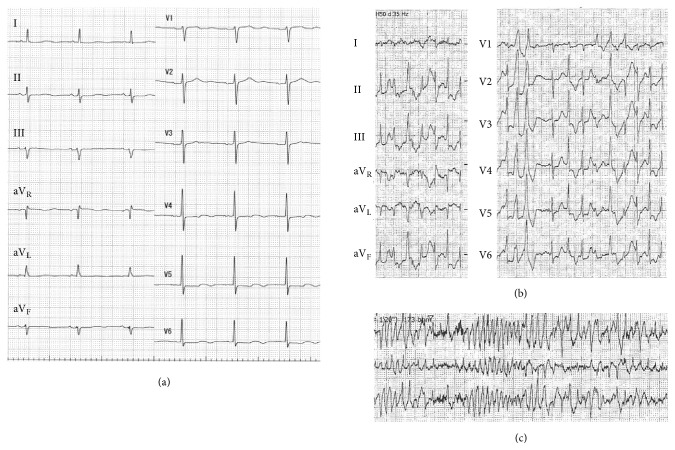
The 12 lead ECG in the patient. (a) The HR was 60bpm. Left axis deviation and enlarged U-waves were noted. An exercise test demonstrated polymorphic ventricular extrasystoles (b) which progressed to sustained polymorphic ventricular tachycardia (c).

**Figure 2:**
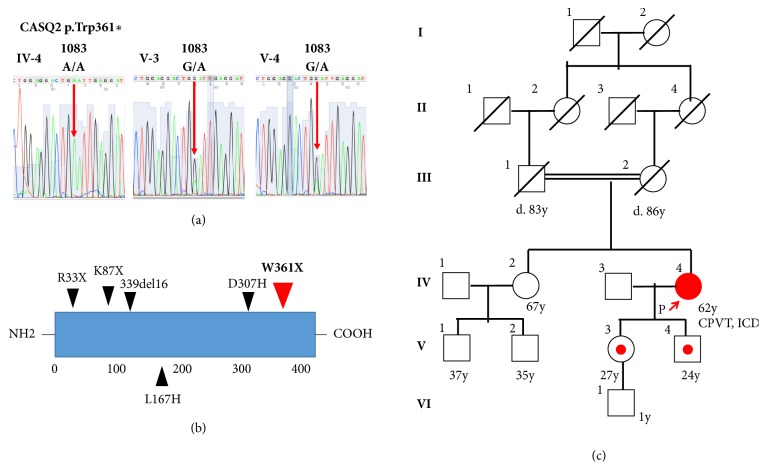
(a) Genetic characteristics of the proband and offspring. The result of DNA sequencing in the index patient (IV-4) demonstrating a homozygous single G to A nucleotide substation at position 1083 of the* CASQ2* gene leading to a Trp361*∗* (=W361X) stop-codon mutation. A heterozygous mutation was identified in her daughter (V-3) and son (V-4). (b) Schematic topology of* CASQ2*, displaying the putative location of the W361X mutation (red arrow). (c) Pedigree of the index family. The proband is marked by an arrow. The results of the* CASQ2* genetic screen were performed on the index patient and her children at an age of 27 and 24, indicating a heterozygous mutation (c.1038 C/G) in the* CASQ2* gene.

A 62-year-old female was referred to our hospital for a genetic evaluation. She had suffered from recurrent syncopal episodes since her childhood. Syncope, which lasted 2 to 3 minutes, developed repeatedly while she was playing table tennis or was distressed during earthquake attacks. Initially, she was diagnosed with epilepsy and was treated by antiepilepsy drugs, which were not effective for preventing her syncope. At the age of 60, she was referred to the cardiology division for an evaluation of bradycardia. However, she refused to undergo an intensive examination and drug therapy for sick sinus syndrome with cilostazol was initiated. She had no family history of sudden death or other cardiac disease. Her physical and neurological examinations were normal. Her ECG at rest exhibited left axis deviation and QT-U prolongation (Figure 1(a)). Late potentials were negative on signal-averaging electrocardiography. Transthoracic echocardiography did not reveal any structural abnormalities. Coronary angiography and an acetylcholine stress test also could not reveal any coronary artery stenosis or coronary vasospasms. The exercise stress testing revealed polymorphic ventricular ectopy (Figure 1(b)), which progressed to polymorphic ventricular tachycardia (Figure 1(c)) accompanied by syncope. She could not undergo electroencephalography or an MRI, including the head and heart, due to her claustrophobia. Based on these findings, she was diagnosed with CPVT. She was implanted with a dual chamber implantable cardioverter defibrillator prior to the prescription of a *β* blocker due to a previous 13 second episode of sinus arrest on the Holter ECG. Afterwards, she was started on bisoprolol and experienced no further syncopal episodes. Upon the patient's request, a genetic evaluation for CPVT was performed. Comprehensive genetic testing was initiated using the TruSight One (Agilent Technologies, Santa Clara, CA) sequencing panel, which targets 4813 genes known to be associated with clinical phenotypes. Genetic testing revealed a homozygous c.1083 G>A/p.Trp361 *∗* stop-codon variant (W361X) in the* CASQ2 *gene (NM_001232.3). This variant was validated by direct capillary sequencing (Figure 2(a)). The W361X variant resulted in the termination of the CASQ2 protein at the C-terminal region (Figure 2(b)) and was reported as pathogenic mutation for CPVT. No other variant was detected in any of the other arrhythmia-related genes. After this homozygous mutation was identified, we further questioned the patient and discovered her parents were consanguineous (Figure 2(c)). She had two asymptomatic children aged 27 and 24. We also conducted genetic testing on them. Both her children carried a heterozygous W361X mutation, which was identical to that of the index patient. These genetic findings coincided with the autosomal recessive trait of a* CASQ2* inheritance. We could not perform a genetic evaluation on the other family members.

## 3. Discussion

CPVT is highly lethal if untreated. Approximately 30% of those patients experience cardiac arrest and 80% experience syncope. Sudden death may be the first onset of the disease. The diagnosis of CPVT is established in a patient with a structurally normal heart, normal resting ECG, and a bidirectional tachycardia or polymorphic VT on the exercise stress test [1]. Identification of heterozygous mutations in* RyR2* or homozygous/compound heterozygous mutations in* CASQ2* can also establish the diagnosis.

We identified a homozygous* CASQ2* mutation in a Japanese CPVT patient. This W361X mutation was located at the C-terminal region of CASQ2. The homozygous mutation of the* CASQ2* gene was first identified in the Bedouin tribe in 2001 [4]. The* CASQ2* mutation accounts for approximately 1% of the CPVT cases, which is a lower rate than that of the* RyR2* mutation identified in 65% of the CPVT cases [3]. It seems rare to form a homozygous mutation in a cohort without a high consanguinity rate. In Japan, only one compound heterozygous* CASQ2* mutation was reported to be causative of CPVT [2]. Patients with the* CASQ2* homozygous/compound heterozygous mutation are known to have a higher rate of sudden death, which affects the patient at an earlier stage than that in those with an* RyR2* mutation [5]. Mutations in CPVT patients were identified in the* RyR2, CASQ2, CALM2, TRD*,* KCNJ2,* and* ANK2* genes [3]. It is suggested that a reduced function of CASQ2, a Ca^2+^ storage protein within the sarcoplasmic reticulum (SR), may lead to increased levels of free Ca^2+^ within the SR, leading to a diastolic leak of Ca^2+^ [3].

In summary, to the best of our knowledge, this is the first report of a recessive* CASQ2* homozygous mutation in Japanese CPVT patients. History taking of a consanguineous marriage in a family is of importance. A genetic analysis using NGS that can detect not only major* RyR2* gene mutations but also minor* CASQ2* mutations is useful.
